# Engineering high levels of saffron apocarotenoids in tomato

**DOI:** 10.1093/hr/uhac074

**Published:** 2022-03-23

**Authors:** Oussama Ahrazem, Gianfranco Diretto, José Luis Rambla, Ángela Rubio-Moraga, María Lobato-Gómez, Sarah Frusciante, Javier Argandoña, Silvia Presa, Antonio Granell, Lourdes Gómez-Gómez

**Affiliations:** 1 Instituto Botánico, Departamento de Ciencia y Tecnología Agroforestal y Genética, Universidad de Castilla-La Mancha, Campus Universitario s/n, Albacete 02071, Spain; 2 Italian National Agency for New Technologies, Energy, and Sustainable Development (ENEA), Biotechnology laboratory, Casaccia Research Centre, 00123 Rome, Italy; 3 Departamento de Ciencias Agrarias y del Medio Natural, Universitat Jaume I, 12006 Castellón de la Plana, Spain; 4 Instituto de Biología Molecular y Celular de Plantas, Consejo Superior de Investigaciones Científicas-Universidad Politécnica de València, Valencia 46022, Spain

## Abstract

Crocins and picrocrocin are high-value hydrophilic pigments produced in saffron and used commercially in the food and pharmaceutical industries. These apocarotenoids are derived from the oxidative cleavage of zeaxanthin by specific carotenoid cleavage dioxygenases. The pathway for crocins and picrocrocin biosynthesis was introduced into tomato using fruit specific and constitutive promoters and resulted in 14.48 mg/g of crocins and 2.92 mg/g of picrocrocin in the tomato DW, without compromising plant growth. The strategy involved expression of CsCCD2L to produce crocetin dialdehyde and 2,6,6-trimethyl-4-hydroxy-1-carboxaldehyde-1-cyclohexene, and of glycosyltransferases UGT709G1 and CsUGT2 for picrocrocin and crocins production, respectively. Metabolic analyses of the engineered fruits revealed picrocrocin and crocetin-(β-D-gentiobiosyl)-(β-D-glucosyl)-ester, as the predominant crocin molecule, as well as safranal, at the expense of the usual tomato carotenoids. The results showed the highest crocins content ever obtained by metabolic engineering in heterologous systems. In addition, the engineered tomatoes showed higher antioxidant capacity and were able to protect against neurological disorders in a *Caenorhabditis elegans* model of Alzheimer’s disease. Therefore, these new developed tomatoes could be exploited as a new platform to produce economically competitive saffron apocarotenoids with health-promoting properties.

## Introduction

Apocarotenoid is widely used within the carotenoid community to refer to all carotenoid cleavage products derived from the oxidative cleavage of carotenoids in a reaction catalyzed by carotenoid cleavage dioxygenases (CCDs) [1]. However, the International Union of Pure and Applied Chemistry (IUPAC) defines apocarotenoids as carotenoids in which the carbon skeleton has been shortened by the removal of fragments from one end or both ends, living outside of this definition compounds such as retinoids [2]. The resulting products of CCDs activities undergo different modifications to render the final active compounds [3] with a broad range of biological functions in all living organisms [1]. In plants, apocarotenoids are involved in plant fitness maintenance and in different aspects of development [4, 5], with similar roles played in animals [6, 7].

**Figure 1:**
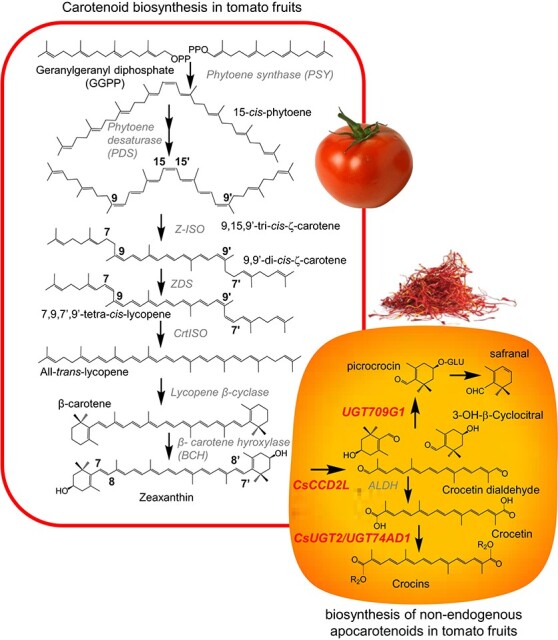
Representative scheme of the carotenoid pathways in tomato and the crocins and picrocrocin pathways from saffron that were introduced into the tomato fruit. Introduced enzymes are highlighted in red. Z-ISO, ζ-carotene isomerase; ZDS, ζ-carotene desaturase; CRTISO, carotene isomerase; ALDH, aldehyde dehydrogenase. HTCC (hydroxy-2,6,6-trimethyl-1-cyclohexen-1-carboxaldehyde).

Carotenoid biosynthesis in plants is catalyzed by nuclear-encoded enzymes that are imported to the plastids [3]. Carotenoids are generated from five-carbon isoprenoids, isopentenyl diphosphate (IPP), and dimethylallyl diphosphate (DMAPP) [3]. Three IPP react with one DMAPP molecule to generate geranylgeranyl diphosphate (GGPP), which is also the precursor for other plastidial isoprenoids. Two GGPP molecules are combined to produce the first pathway compound, 15-cis-phytoene (Fig. 1), in a reaction catalyzed by phytoene synthase (PSY), and is considered the main point of control for the carotenoid biosynthetic pathway [8]. This is followed by multiple steps of desaturations and isomerizations leading to all-trans-lycopene formation, which is the major carotenoid in ripe tomatoes (Fig. 1). Further cyclization and hydroxylation reactions result in lutein and zeaxanthin (Fig. 1). In plants, zeaxanthin and lutein have several functions, including photoprotection, detoxification of reactive oxygen species and other radicals, and functional and structural integrity maintenance of biological membranes [9]. Furthermore, zeaxanthin acts as a precursor for the biosynthesis of apocarotenoids as crocins and picrocrocin in saffron, buddleja, and gardenia, and ABA biosynthesis starts with the epoxidation of zeaxanthin [9–11].

Crocins and picrocrocin are water-soluble apocarotenoids, which accumulate in large amounts in the stigma of saffron (*Crocus sativus*), and at lower levels in buddleja and gardenia [12]. Saffron has been known since ancient times for its health promoting properties [13, 14], for which these characteristic apocarotenoids are partially responsible. Saffron is mainly used as a spice and referred to as “red gold” due to its high economic value, which is a direct consequence of the specific characteristics of its cultivation and management [15]. Between 120 000 and 200 000 flowers are needed to produce 1 kg of dried saffron stigma threads, which equates to 370–470 h of work. Consequently, the process is very labor-intensive as well as risky since it is highly dependent on environmental conditions, leading to high costs and restricting the utilization of saffron by other industrial sectors.

Crocins are responsible for the strong red color of saffron, and picrocrocin is associated with its bitter taste, and it is the precursor of safranal, the compound responsible for the peculiar aroma and flavor [12, 15]. These saffron apocarotenoids are derived from the cleavage of zeaxanthin at 7,8:7′,8′ positions, in a reaction catalyzed by a specific carotenoid cleavage dioxygenase enzyme (CsCCD2L) [16, 17]. As result of this cleavage reaction, two molecules of 6,6-trimethyl-4-hydroxy-1-carboxaldehyde-1-cyclohexene (HTCC) and one molecule of crocetin are produced (Fig. 1). These compounds acquired water-solubility by glucose molecules added to their skeletons by the action of glucosyltransferases; CsUGT2, which added glucose to the crocetin molecule, and UGT709G1 that is involved in the glucosylation of HTCC producing picrocrocin [18, 19] (Fig. 1).

With the advances of metabolic engineering and its progression into synthetic biology, it is now possible to extend the carotenoid pathway in other hosts, for the low-cost production of rare metabolites, such as saffron apocarotenoids, with a global market size valued at 881.7 million USD in 2019. Tomato (*Solanum lycopersicum*) is a popular and highly consumed food worldwide, being used as a model crop for biotechnological applications [20]. In this study, tomato was selected for the introduction of the saffron pathway because the fruit accumulates high levels of carotenoids as substrates, and there are well established downstream processes, including concentrates and juices for human consumption [21]. The apocarotenoids of saffron might provide the characteristic flavor and aroma of the saffron spice, and their solubility will enhance the nutraceutical properties of tomato juice. By using a combinatorial approach in which *CsCCD2L* was introduced with *CsUGT2* and *CsUGT709* under the control of different promoters, a maximum crocins accumulation in line O1 11 up to 14.48 mg/g DW was reached, which has been the highest level reported for saffron apocarotenoids in heterologous systems as a result of transient or stable expression. Additionally, apocarotenoid-enriched tomatoes showed high antioxidant activity and exerted high effectiveness in Alzheimer’s disease reduction, as revealed by using a *C. elegans* model.

Overall, this study demonstrates the potential and feasibility of using tomatoes as a biotechnological platform for the simultaneous production of pro-nutritional lipophilic and rare high-valuable hydrophilic apocarotenoid compounds that are beneficial for both industry and human health.

## Results

### Generation of vectors for ectopic expression of the saffron apocarotenoid pathway in tomato transgenic lines

Initially, three different promoters were selected and evaluated for saffron gene expressions in tomato (*S. lycopersicum* cv. Moneymaker (MM)). The pE8 and p2A11 are fruit specific promoters from tomato (see tissue-specific expression of the corresponding genes in Supplementary data Fig. S1), whose activity could result in high levels of accumulation of the desired metabolites without yield penalties, because fruit set, development, and ripening are mostly completed at the time of their activation [22, 23]. These promoters were used for *CsCCD2L* expression in constructs O1 (pE8), O2 (pE8), and O4 (p2A11), and for *CsUGT2* expression in construct O2 (p2A11) and O4 (pE8). The constitutive CaMV 35S promoter was used to drive the expression of all the genes in construct O3, of *CsUGT2* and *UGT709G1* in construct O1, of *UGT709G1* in construct O2, and of *UGT709G1* in construct O4. Four different binary vectors were created and used for the tomato transformations (Supplementary data Fig. S2). The tomato variety MM was selected for *Agrobacterium*-mediated transformation as it is widely used in tomato genetic studies. Transgenic lines were obtained for all constructs: O1 (20 lines), O2 (3 lines), O3 (5 lines), and O4 (12 lines), but not all the lines survived after transfer to the soil and adaptation to the greenhouse conditions. Six lines able to produce fruits were selected, and the expression levels of the introduced transgenes were confirmed by qRT-PCR analysis (Supplementary data Fig. S3). Seeds were collected from fruits of the primary transgenic lines. Transformed plants carrying the construct O3 failed to produce any viable seeds (Supplementary data Fig. S4). The fruits of these plants showed a striking orange color, and differences in the coloration of its flower parts (Supplementary data Fig. S4). All the other primary transgenic lines presented flowers and fruits with no obvious phenotypic differences compared to the wild-type (WT) (Supplementary data Fig. S5). Interestingly, these latter lines produced fruit with viable seeds, which showed differences in color appearance in the following generations (Supplementary data Fig. S6).

In addition, all the transgenic lines showed high antioxidant activity based on their radical scavenging activity (Supplementary data Fig. S7). Compared with the fruit of WT, the antioxidant capacity of the fruits of these transgenic lines was about 3 to 7-fold higher.

### Apocarotenoid evaluation of fruits from transgenic plants

**Figure 2:**
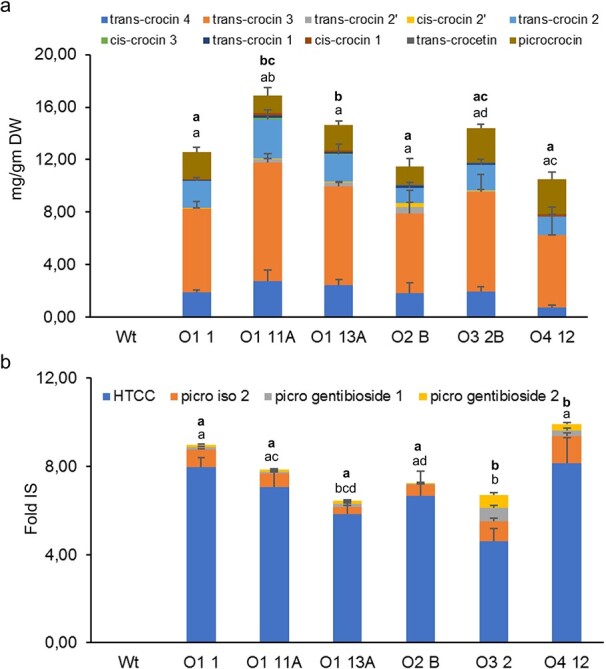
Metabolic engineering of saffron apocarotenoid in tomato fruits. **a** Crocetin, crocins and picrocrocin contents in the WT and transgenic lines. **b** HTCC, and picrocrocin derivatives contents in the WT and transgenic lines, referred to the internal standard formononetin (IS). Two Br + 10 fruits per plant, and 3 plants per line, were analyzed. Standard error (SD) represents three determinations. Different letters represent statistically significant differences of means, of transgenic lines, according to factorial analysis of variance (ANOVA) (P ≤ 0.05) (Tukey test). In **a**, above letters in bold correspond to differences in total crocins content, and letters below correspond to differences in picrocrocin content. In B, above letters in bold correspond to differences in total picrocrocin derivatives content, and letters below correspond to differences in HTCC content.

Fruits from lines O1, O2, O3 and O4, were evaluated for the presence of saffron apocarotenoids (Fig. 2a and b). The analyzed lines showed the presence of all the apocarotenoids that are characteristic of saffron at variable levels. For crocetin and crocins accumulation, lines O1 1, O1 11A, O1 13A, O2 B, and O3 2B, showed high levels, including *trans*-crocin 3 [(crocetin-(β-D-gentiobiosyl)-(β-D-glucosyl)-ester)], which was preferentially accumulated (Fig. 2a). More in detail, by comparing the different constructs using an ANOVA plus pairwise Tukey’s t-test analysis, line O1 11A turned to be the best performer in terms of total crocins levels (Fig. 2a). Picrocrocin levels were higher in lines O3 B and O4 12 (Fig. 2a). In addition, HTCC, other picrocrocin derivatives and isomers, were also analyzed in these fruits (Fig. 2b). These apocarotenoids were detected in all the lines analyzed, and were, as expected, absent in the fruits of the WT plants.

The content of ABA and its catabolite and storage forms (7-OH-ABA and ABA-glucose ester, respectively) were also analyzed in these lines. Overall, the levels of these metabolites were reduced in the transgenic fruits (Supplementary data Fig. S8), suggesting that zeaxanthin conversion to crocetin dialdehyde impairs ABA biosynthesis [10], but without affecting the development or viability of the seeds with the exception of the O3 lines.

### Levels of carotenoids and related compounds in WT and transgenic tomato fruits

Transgenic and control fruits were subjected to carotenoid extraction and HPLC-APCI-HRMS analyses to determine their carotenoid profiles (Table 1).

**Table 1:** Carotenoids identified and quantified in ripe fruits of transgenic lines and WT. Results are presented as the ratio of transgenic lines vs WT.

carotenoid	WT AVG ± SD	O1 1 AVG ± SD	O1 11A AVG ± SD	O1 13A AVG ± SD	O2 B AVG ± SD	O3 2B AVG ± SD	O4 12 AVG ± SD
lutein	1 ± 0.12	**0.13 ± 0.13**	NA	NA	NA	**0.18 ± 0.04**	**0.02 ± 0.00**
phytoene	1 ± 0.22	1.59 ± 0.34	0.65 ± 0.17	0.58 ± 0.13	0.52 ± 0.16	**0.54 ± 0.08**	0.84 ± 0.12
phytofluene	1 ± 0.26	1.46 ± 0.32	0.71 ± 0.33	**0.32 ± 0.05**	**0.22 ± 0.01**	0.71 ± 0.13	0.92 ± 0.22
α-carotene	1 ± 0.20	0.54 ± 0.39	NA	NA	NA	NA	NA
β-carotene	1 ± 0.24	**0.25 ± 0.05**	**0.03 ± 0.02**	**0.01 ± 0.00**	**0.02 ± 0.01**	**0.03 ± 0.00**	**0.01 ± 0.00**
lycopene	1 ± 0.11	**0.30 ± 0.03**	**0.12 ± 0.02**	**0.03 ± 0.01**	**0.05 ± 0.01**	**0.06 ± 0.02**	**0.06 ± 0.01**
Total carotenoids	1 ± 0.13	**0.50 ± 0.08**	**0.19 ± 0.04**	**0.11 ± 0.02**	**0.11 ± 0.03**	**0.14 ± 0.02**	**0.17 ± 0.03**

Two Br + 10 fruits per plant, and 3 plants per line, were analyzed. Standard error (SD) represents three determinations. Values in bold indicate a significant difference with respect to WT (P < 0.005). NA: not applicable.

In general, the carotenoid content levels in all the transgenic fruits were remarkably reduced compared to that in the WT. Different carotenoid profiles were found in the ripe fruits of the WT, and in the transgenic lines, depending on the transgenic event. The most striking differences were related to lycopene, β-carotene, and lutein content levels. WT fruits accumulated up to 29-fold and 71-fold higher amounts of lycopene and β-carotene, in comparison with line O1 13A, respectively. Lutein and α-carotene levels were reduced or undetectable in the transgenic lines (Table 1).

**Figure 3:**
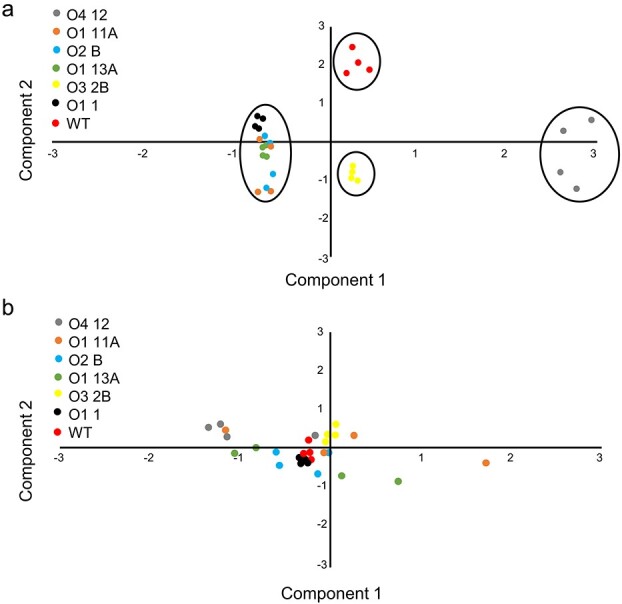
Principal Component Analysis (PCA) of untargeted metabolites measured in fruits of WT and transgenic lines. **a** Untargeted polar and **b** nonpolar principal component analysis score plots showing the metabolites extracted from the (**a**) polar and (**b**) nonpolar lines of the 6 transgenic lines and the WT. Principal components 1 and 2 have been represented. Biological replicates in the score plots are shown with the same color within each line.

### Untargeted analysis of polar and non-polar metabolites

The untargeted metabolomic analyses identified 70 polar and 58 non-polar differentially accumulated metabolites (DAMs) in the analyzed samples, besides apocarotenoids and carotenoids (Fig. 2, Table 1 and Supplementary data Table S3A-B). In Fig. 3a, the principal component analysis (PCA) of the polar metabolites clearly kept the WT separated from the transformed lines. However, the apocarotenoid-accumulating lines could be clustered according to the construction: component 1 allowed for the clustering of all O1 lines, with O3 2B being located further away from the O1 group, while line O4 12 was mapped outside of the 95% confidence interval and performed as an outlier in the analyzed collection. In contrast, the PCA of the non-polar metabolome did not enable a clear discrimination of the WT and transformed lines (Fig. 3b), although component 1 highlighted line O4 12 as more diverged when compared to the WT and the other transformants in terms of metabolite accumulation. Thus, the untargeted global metabolomic approach revealed that it is the polar metabolome rather than the non-polar metabolome that is most affected in this metabolic engineering project. When the annotation pipeline was used, a large number of compounds (44 out of 70, and 25 out of 58 for, respectively, polar and non polar metabolome) was identified and validated (Supplementary data Table 3A-B; for more details, see Materials and Methods): overall, and besides a series of stochastic changes, it was not possible to define clear and conserved trends at polar metabolome level in all the crocins-accumulating lines compared to the Wt, albeit O1 and O4 lines were characterized by, respectively, a high number of negative and positive alterations (Supplementary data Table 3A), and with line O3–2 in the middle in terms of over- and down-accumulated compounds. Notable exceptions were represented by a significant increase in chorismic acid, a reduction in p-Hydroxycinnamic acid and dl-Tryptophan and, in 8 out of 9 transgenic lines, in L-Phenylalanine and Tyramine (Supplementary data Table 3A); on the contrary, a more variegated biochemical phenotype was found in the non polar metabolome, with lines O3–2 and O4–8 having a general over-accumulation in most of detected DAMs, but with a single unidentified compound (m/z 473.28207 in APCI(−)-MS) showing higher levels in all lines under study (Supplementary data Table 3B).

### Volatile compound evaluation in the fruits from transgenic plants

Analyses of the volatile compounds in selected lines resulted in the identification of 14 different volatiles derived from carotenoids. The abundance of these apocarotenoids is shown in Table 2.

**Table 2:** Volatiles emitted by ripe fruits of transgenic lines and WT. Results are presented as the ratio of transgenic lines vs WT.

Compounds	O1 11A	O1 13A	O1 1	O4 12	WT
6-methyl-5-hepten-2-one	1.30 ± 0.08	0.26 ± 0.04	1.90 ± 0.07	1.57 ± 0.05	1 ± 0.13
2.6-Dimethyl-1.3.5.7-octatetraene. E.E-	**85.54 ± 3.02**	**58.18 ± 5.70**	1.34 ± 0.26	**33.23 ± 4.24**	1 ± 0.21
2-Cyclohexen-1-ol	1.21 ± 0.04	1.22 ± 0.05	**0.07 ± 0.00**	**2.85 ± 0.36**	1 ± 0.12
Benzene. 1.2.3.5-tetramethyl (isodurene)	**27.95 ± 1.66**	**28.02 ± 1.71**	0.27 ± 0.00	**97.10 ± 7.89**	1 ± 0.25
Methyl salicylate	1.3 ± 0.37	4.72 ± 1,16	0.73 ± 0.21	1.71 ± 0.54	1 ± 0.30
beta-cyclocitral	20.63 ± 3.51	1.70 ± 0.86	2.01 ± 0.08	1.95 ± 0.02	1 ± 0.56
Geranial	0.95 ± 0.01	0.22 ± 0.00	1.65 ± 0.02	1.37 ± 0.02	1 ± 0.10
Ethyl salicylate	0.04 ± 0.00	0.14 ± 0.00	0.05 ± 0.00	1.20 ± 0.06	1 ± 0.15
beta-damascenone	0.32 ± 0.05	0.17 ± 0.00	0.70 ± 0.00	0.55 ± 0.04	1 ± 0.01
4-(2.6.6-Trimethylcyclohexa-1.3-dienyl)but-3-en-2-one	**3.22 ± 0.25**	5.58 ± 0.38	0.81 ± 0.00	2.25 ± 0.01	1 ± 0.11
Geranylacetone	1.14 ± 0.27	0.3 ± 0.00	2.6 ± 0.32	1.31 ± 0.02	1 ± 0.29
beta-ionone	0.65 ± 0.28	0.23 ± 0.10	1.42 ± 0.28	1.12 ± 0.11	1 ± 0.07
*safranal	**31.23 ± 1.25**	**27.48 ± 1.46**	**1.00 ± 0.03**	**30.87 ± 2.25**	NA
*safranal isomer	**40.21 ± 1.97**	**26.82 ± 1.82**	**1.00 ± 0.02**	**47.18 ± 3.52**	NA

Two Br + 10 fruits per plant, and 3 plants per line, were analyzed. Standard error (SD) represents three determinations. Values in bold indicate a significant difference with respect to WT (P < 0.005). NA: not applicable. *These compounds were undetectable (NA) in WT fruits and the ratio was calculated as transgenic lines vs line O1 1, as was the line that showed the lowest levels of these compounds among all the analyzed lines.

Safranal and β-cyclocitral are the result of the 7,8:7′,8′ cleavage activity over zeaxanthin and β-carotene, respectively. β-cyclocitral is a minor constituent of the tomato fruit volatile profile. Its production was increased in the transgenic lines, some of which showed over 20-fold higher levels. In turn, safranal constitutes a novel compound in the tomato volatile blend and was detected in all the transgenic lines. Another volatile apocarotenoid of similar structure was detected only in the transgenic lines. It was tentatively identified as a C_10_ cyclic apocarotenoid alcohol and could be derived from the cleavage of lutein in the 7′,8′ bond close to the α-ring.

With the only exception being the already cited β-cyclocitral, the other known apocarotenoids naturally produced in the tomato fruit were either linear, such as 6-methyl-5-hepten-2-one, geranial, and geranylacetone_,_ or cyclic, such as β-damascenone and β-ionone, and showed similar levels in the WT and the transgenic lines.

### Evaluation of the crocins in T2 fruits from selected lines

Fruits from selected lines were analyzed for their total crocins and picrocrocin content. The fruits showed no color changes in their peels compared to WT fruits (Supplementary data Fig. S9a). However, when cut in half, an orange coloration could be observed in their internal part (Supplementary data Fig. S9). Tomatoes were crushed and the serum juice obtained after centrifugation. This serum from the transgenic lines showed a yellow to orange color in comparison to the almost colorless extract from the WT fruits (Supplementary data Fig. S9b). Crocins and picrocrocin were detected in all the yellow-orange serum juices, but not in the control tomato serum juice (Table 3).

**Table 3:** Content of saffron apocarotenoids in fruits and serum juice of WT and transgenic lines

Plants	fruit		juice	
	Crocins mg/g DW	Picrocrocin mg/g DW	Crocins g/L	Picrocrocin g/L
WT	NA	NA	NA	NA
O1 1	9.96 ± 0.21^c^	2.23 ± 0.07^c^	0.12 ± 0.01^c^	0.02 ± 0.00^a^
O1 11A	14.48 ± 0.18^a^	2.42 ± 0.06^b^	0.18 ± 0.02^a^	0.02 ± 0.00^a^
O1 13A	12.86 ± 0.16^b^	1.72 ± 0.13^d^	0.15 ± 0.01^b^	0.02 ± 0.00^a^
O2 B	7.32 ± 0.10^e^	1.49 ± 0.22^e^	0.06 ± 0.03^e^	0.01 ± 0.00^b^
O4 12	8.77 ± 0.23^d^	2.92 ± 0.09^a^	0.10 ± 0.00^d^	0.02 ± 0.00^a^

Two Br + 10 fruits per plant, and 3 plants per line, were analyzed. Standard error (SD) represents three determinations. NA: not applicable. Different letters indicate significant differences among samples (P < 0.05) (one way ANOVA with Tukey’s test).

Fruits with up to 14.48 mg/g of dry weight of crocins were obtained from fruits in the original line O1 11A (Table 3). This line showed the highest levels of crocins in the serum juice (Table 3) and was further evaluated for the accumulation of crocins and picrocrocin in fruits at different developmental stages (Supplementary data Fig. S10). Crocins and picrocrocin were not detected in the mature green fruit, and very low levels were already detected in the breaker stage compared with fruits in the mature red stage (Supplementary data Fig. S10a). This line was also evaluated for the expression levels of several carotenogenic genes such as *PSY1* (fruit-specific phytoene synthase gene), *PSY2*, *LCYB* (lycopene β-cyclase) and *BCH1* (β-carotene hydroxylase). Significant differences were observed for *PSY2* in O1 11A fruits compared with WT fruits (Supplementary data Fig. S10b), while the expression pattern of the other genes were similar in the analyzed fruits (Supplementary data Fig. S10b). This finding is of particular interest, since in tomato fruit *PSY1* is the well-known master regulator of carotenoid biosynthesis. In this context, the up-regulation of *PSY2* would suggest an increase in the phytoene flux in the pathway involving another isoform with respect to the main one acting in tomato fruit carotenogenesis.

**Figure 4:**
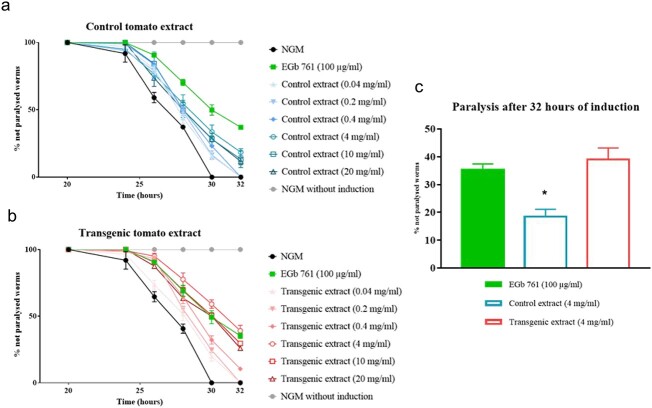
Effects on the paralysis induced in *C. elegans* CL4176 by the different extracts of control and transgenic tomato. **a** Effects of the control tomato extract. **b** Effects of the transgenic tomato extract enriched in crocins. Crocin content in each transgenic extract dilution was 0.1, 0.5, 1, 10, 25 and 50 μg/ml. **c** Comparison of the control and transgenic extracts at 4 mg/ml dilution with EGb 761 (100 μg/ml). NGM: negative control. EGb 761: positive control. * p < 0.05.

An additional experiment was performed to compare the pigmentation power of the dry tomatoes with that of saffron threads in a simulated cooking process. For this experiment, two procedures were followed. In the first one, dry transgenic tomato (0.622 g), the same quantity of tomato WT, and the threads from one flower of saffron (0.002 g), were immersed each in 50 mL of water at 90°C (Supplementary data Fig. S11). In the second procedure, 0.02 g of powdered dry transgenic tomato, WT tomato, and saffron were combined with 5 ml of water (Supplementary data Fig. S11). The diffusion of the crocins in the water was analyzed by HPLC-DAD at 10, 30, and 60 min after the introduction of the samples in the solution (Supplementary data Fig. S11). The sliced dry tomatoes showed a rapid release of crocins compared to the saffron stigmas, even though after 60 min the coloring power of the saffron was much higher. When the powder from the transgenic dry tomatoes was used, the release of the crocins and the coloring power was greater with the cut tomatoes. The results showed the clear coloring power of the engineered tomato, although different crocin kinetics of accumulation in the solution were observed, depending on the material used, and the method of sample preparation. In the tomato fruit, the uniform mixture of the dry powder was more efficient than the sliced one, which clearly affected the diffusion of the crocins in the solution (Supplementary data Fig. S11). Such differences could be due to the presence of the tomato peel, preventing the spread of crocins from the inside, and provide interesting hints about the potential industrial exploitation of these high-crocins tomatoes.

### Effect of tomato extracts on *Caenorhabditis elegans* Alzheimer model

Saffron has neuroprotective effects and several evidences demonstrate that crocin might be a promising target for cognition improvement in AD and stress-related disorders [24]. To evaluate the anti-Alzheimer disease effects of the obtained transgenic tomatoes, extracts from wild-type and transgenic tomato O1 11A, were evaluated using the transgenic *C. elegans* CL4176 strain [25]. Both extracts from wild-type and transgenic tomato, showed a protective effect against worm paralysis in all analyzed concentrations compared with the negative control at the different analysis times (Fig. 4a and b). The most effective concentration in both extracts was 4 mg/ml, corresponding to 10 μg/ml of crocins in transgenic tomato extracts. The same effect was observed at higher concentrations (10 and 20 mg/ml of extract). After 32 hours of paralysis induction through Aβ1–42 expression, the wild-type extract at 4 mg/ml showed a significant lower effect compared with the EGb761 positive control and the transgenic extract at 4 mg/ml. The transgenic extract and the positive control did not show significant differences (Fig. 4c). The transgenic tomato extract that accumulates crocins showed a significantly lower paralysis of *C. elegans* CL4176 with induced Aβ1–42 expression than the control tomato extract. The effect of the transgenic tomato extract was the same at the three highest crocin concentrations tested (10, 25, and 50 μg/ml), and showed no significant differences with the EGb 761 positive control.

## Discussion

Crocins and picrocrocin are major components of saffron, and in addition to their organoleptic properties, they also display therapeutic properties, such as antioxidant, anticonvulsant, neuroprotective, anti-inflammatory, antidepressant, and anti-proliferative activities [26, 27]. In this study, we successfully demonstrated that by engineering tomato fruits to express a series of saffron genes that are involved in the biosynthesis of apocarotenoids in the crocus stigmas, the biosynthesis and accumulation of high levels of crocins and picrocrocin in their fruits occur.

Tomato fruits were revealed to be a good platform for producing the characteristic apocarotenoids of saffron. In ripening tomato fruits, the carotenoid pathway is highly active and, which is why tomato fruits have previously been used to produce different exotic carotenoid compounds, such as ketocarotenoids [28–30]. Whereas the biosynthesis of ketocarotenoids involved a single subcellular location in the cell, the plastid [28], the pathway for crocins and picrocrocin is more challenging, as it requires the participation of two main compartments: the plastid (the source) and the vacuole (the sink), where crocins accumulate [31–33]. The central vacuole functions as a storage compartment for hydrophilic secondary metabolites [34], allowing for the accumulation of huge amounts of metabolites, and thus offering an attractive compartment for the accumulation of polar secondary metabolites of interest.

By expressing the three genes necessary to produce crocins and picrocrocin in tomato, we obtained high levels of these valuable metabolites, indicating that these genes were sufficient to confer tomato with the ability to produce them. This resulted in decreased lycopene levels in ripe tomato. Despite this, zeaxanthin was still not a major carotenoid in the tomato fruit [35]. This indicated that CsCCD2L was able to intercept the fruit metabolic flux diverting the primary isopentenyl diphosphate precursor towards the production of apocarotenoids in these fruits; reducing the available pool for the biosynthesis of other isoprenoid compounds; and decreasing the accumulation of lycopene, β-carotene, and α-carotene. Similarly, such effects have been observed in *N. benthamiana* leaves, where crocins were produced via the expression of *CsCCD2L* using a virus-driven system [19, 33]. The expression of *CsCCD2L*, *UGT74AD1,* and *UGT709G1* in tomato allowed for the accumulation of notable amounts, up to 14.48 ± 0.18 mg of crocins and 2.42 ± 0.06 mg of picrocrocin per gram (DW). The variation observed among the analyzed lines could result from the positional effects of the inserted transgenes or as a consequence of the copy number of the transgenes. The amount of crocins obtained here was much higher than in previous studies reported using *Agrobacterium tumefaciens*-mediated transient expression of *CsCCD2L* in *N. benthamiana* leaves, alone or in combination with *UGT709G1* (30.5 μg/g DW of crocins) [19]; and in leaves of adult *N. benthamiana* using a recombinant virus that expressed *CsCCD2L* (2.18 mg/g DW of crocins) [33]. Additionally, the described approach has the advantage of using stably transformed tomatoes rather than *N. benthamiana* leaves which have to be repeatedly transiently transformed, thus offering an easy and constantly available source of valuable apocarotenoids. The quantity of crocins obtained from the tomatoes was 10 to 20 times lower than that reported for saffron from different geographical origins [36]. Comparison of our data with the production of other metabolites in tomatoes, such as ketocarotenoids (3 mg/g DW) [37], anthocyanins (5 mg/g DW) [22], resveratrol (5–6 mg/g DW) [23], and betacyanin (50 mg/L) in tomato juice [38], demonstrates the feasibility of engineering economically important metabolic pathways in tomato fruits with good yields for compound interests. Further, agronomic management could have a significant impact in the obtained yield of crocins in the fruits, as carotenoid content could be increased in tomato by reduction in irrigation, as noted in other studies [39], increasing the substrate pool.

Saffron experiences a high degree of fraudulent production globally, due to it high market value [40]. We estimate that the saffron apocarotenoids produced in these tomato fruits could provide an approximate 150-fold cost saving, as presently the production costs for saffron spice are in the range of 3000–6000 USD per kilogram, assuming a production cost for tomato material containing a kilogram of saffron apocarotenoids of around 32 USD.

At the qualitative level, the crocin profiles were slightly different than saffron stigma profiles, where trans-crocin 4, followed by trans-crocin 3 are the major crocins detected [41]. In contrast, in our transgenic tomato fruits, although these two types of crocins were still the major compounds detected, the order was reversed. In *N. benthamiana* leaves expressing only *CsCCD2L*, the major crocin species were the trans-crocin 3, and trans-crocin with two glucose molecules [33]. The participation of endogenous and promiscuous UGT enzymes in tomato and tobacco with different substrate affinities and specificities compared to those of saffron were observed; the presence of glucosidases in these heterologous systems could influence crocins molecules by changing their qualitative relationships [42]. This could explain as well the lower picrocrocin levels in these tomato lines (2.92 mg/g DW) which was 2.5 times lower than those detected in tobacco [19, 33]. In saffron, levels of picrocrocin can reach from 16 to over 42 mg/g DW depending on their method of preparation [43]. In the fresh saffron stigmas, safranal levels are very low [41], and not detected in the stigma-like structures generated *in vitro* [44]. However, their levels were considerably increased (16–41 mg/g) after the processing of the spice due to the thermal degradation of picrocrocin [45]. Safranal was detected in all the transgenic fruits, together with a safranal-related compound (probably derived from the cleavage of lutein). Notably, lutein displayed the highest decrease, likely due to the ability of CsCCD2L to use it as an alternative substrate [17, 33].

Our results show that the total carotenoid concentrations in the transgenic fruits were much lower than in the WT tomato. The steady-state concentration of the carotenoids is controlled by the flux through the biosynthetic pathway, by several mechanisms that determine the storage capacity of the plastid, and the degradation processes [46]. In this context, the general reduction in carotenoids could be explained by the flux diversion towards apocarotenoid synthesis. Zeaxanthin in normal fruit is metabolized to violaxanthin, which is the precursor for ABA biosynthesis [47]. In this study, due to the conversion of zeaxanthin to crocins and picrocrocin, the levels of ABA in the transgenic fruits were remarkably reduced compared to the WT. However, this reduction did not affect seed production in the lines where *CsCCD2L* was under fruit-specific promoters. Only line O3 was unable to produce seeds, which suggested that constitutive *CsCCD2L* expression was accompanied by detrimental effects during seed development. We evaluated, by untargeted non volatile and targeted volatile metabolomics, the presence of additional alterations in crocins-accumulating lines compared to the WT; overall, a set of unspecific changes was observed, thus proving saffron apocarotenoid synthesis in fruit has a limited impact on fruit metabolism. Interestingly, chorismic acid and dl-tryptophan and phenylalanine showed, respectively, higher and lower levels in transgenic lines versus WT lines: the former takes part in the plastidial shikimate pathway, while the latter’s are synthesized in the frame of its catabolism. These changes are cryptic since no direct connections have been described at fruit level between chorismic acid and apocarotenoid synthesis.

Tomatoes are a good source of health-promoting nutraceuticals and micronutrients. Total antioxidant activity in the transgenic lines (with reduced liposoluble carotenoids and high water-soluble apocarotenoids) was much higher than in the WT. It has been reported that saffron has higher antioxidant activity levels compared to tomato [48]. Moreover, crocins are more bioaccessible than lipophilic carotenoids, thus the engineered tomatoes for crocins and picrocrocin production could substantially increase their generally low hydrophilic antioxidant available levels [49]. In addition, the protective effects of crocins against chronic stress-induced oxidative damage and their anti-inflammatory effects have been widely reported [50], and are due to their ability to modulate redox status of organisms. Further, by using transgenic *C. elegans* CL4176 as a model for Alzheimer, we showed a significant delay of Aβ induced paralysis in worms treated with the serum extracts of transgenic tomatoes. Therefore, the presence of saffron apocarotenoids will help to enhance the health properties of these tomatoes, which can be consumed either fresh or as processed products such as juices, soups, and sauces [20]. In addition, crocins and picrocrocin can also simply be extracted from the pressed juice of the tomato and used for the affordable production of these compounds as pharmaceuticals in medicine or as colorants for the feed, food, and beverage industries.

## Experimental procedures

### Plant material and growth conditions

Tomato (*S. lycopersicum* cv. Moneymaker (MM)) was used as the wild type and the genetic background for all plant transformations. Plants were treated and cultivated as previously described [51]. Tomato fruits were mainly collected at the ripe stage and, once separated from the seeds, were cut into pieces, and immediately frozen in liquid nitrogen until further analyses.

### Vector construction

A series of plasmids were created with different promoter combinations to evaluate their ability to engineer the saffron apocarotenoid pathway in tomatoes (Fig. S2). Three promoters, pE8, p2A11, and p35S, were selected to drive the expression of the saffron genes in tomato. The Goldenbraid strategy was followed to construct the vectors [52, 53]. Briefly, the complete open reading frames (ORF) of *CsCCD2L*, *CsUGT2* (*UGT74AD1*), and *UGT709G1* were domesticated from the sequences provided in previous research [16, 18, 19] by removing the *Bsm*BI and *Bsa*I restriction sites present in the original sequence using the primers listed in Supplementary data Table S1. The products were cloned in the level 0 vector pUPD2 of the Goldenbraid modular cloning system. The resulting plasmids pUPD2-CsCCD2L, pUPD2-CsUGT2, and pUPD-CsUGT709G1 were then used to construct 4 recombinant binary vectors as follows: O1 = pDGB3α1[p35S:UGT2:T35S-p35S:UGT709G1:T35S-pE8:CCD2L:T35S-pNos:Hyg:T35S], O2 = pDGB3α1[p2A11:UGT2:T35S-p35S:UGT709G1:T35S-pE8:CCD2L:T35S-pNos:Hyg:T35S], O3 = pDGB3α1[p35S:UGT2:T35S-p35S:UGT709G1:T35S-p35S:CCD2L:T35S-pNos:Hyg:T35S], and O4 = pDGB3α1[pE8:UGT2:T35S-p35S:UGT709G1:T35S-p2A11:CCD2L:T35S-pNos:Hyg:T35S].

### Tomato transformations

Plasmids were transferred to *A. tumefaciens* LBA4404 strain by electroporation and then used for tomato stable transformation into *S. lycopersicum* (var. Moneymaker), as described previously [54]. Transgenic plants were selected on hygromycin selection media, and the genomic DNA was further verified by PCR using primers targeting, *CsCCD2L*, *UGT709G1* and *CsUGT2* genes (Supplementary data Table S2), to check for the presence of the corresponding genes. All in vitro steps were carried out in a long-day growth chamber (16 h light/8 h dark, 24°C, 60–70% humidity, 250 μmol/m2/s). Positive transgenic tomato plants were transferred to soil and grown in a greenhouse with a 14/10 h day/night photoperiod at 22°C.

### Apocarotenoid and carotenoid analyses

Polar and nonpolar metabolites were extracted from 50 mg and 5 mg of lyophilized ripe fruit tissues (sampled 10 days after color turning, Br + 10), respectively. For the polar metabolite analyses (crocins and picrocrocin), the tissue was extracted in cold 75% methanol. Apolar metabolites (crocetin, HTCC, and carotenoids) were extracted with 50:50 methanol:CHCl_3_. Polar and apolar fractions were analyzed by HPLC-DAD-HRMS and HPLC-DAD as previously described [19]. Metabolites were identified using co-migration with standards, by matching the UV spectrum of each peak against that of a standard, when available, on the basis of literature data, and *m/z* accurate masses, as reported in the Pubchem database (http://pubchem.ncbi.nlm.nih.gov/) for monoisotopic mass identification or using the Metabolomics Fiehn Lab Mass Spectrometry Adduct Calculator (http://fiehnlab.ucdavis.edu/staff/kind/Metabolomics/MS-Adduct-Calculator/) in the case of adduct detection. Pigments were quantified by integrating the peak areas that were converted to concentrations by comparison with the standards and as reported previously [33].

### Volatile analyses

Volatile compounds were captured by HS-SPME and analyzed by GC–MS. Analyses of the volatile compounds were performed similarly to a previously described process [55]. Roughly, 500 mg of the resulting powder from tomato fruits was introduced into a 15 mL glass vial and incubated at 37°C for 10 min in a water bath. After, 0.5 mL of EDTA 100 mM, pH 7.5 and 1.1 g of CaCl_2_.2H_2_O were added, mixed and sonicated for 5 min. 1 mL of the mixture was transferred to a 10 mL vial with silicon/PTFE septum and analyzed within 12 hours. Volatile extraction was performed with a CombiPAL autosampler. Desorption was performed at 250°C for 1 min. Chromatography was performed on a DB-5 ms (60 m, 0.25 mm, 1.00 μm) capillary column with helium at a constant flow of 1.2 mL/min. The GC interface and MS source were at 260°C and 230°C, respectively. Oven conditions were 40°C for 2 min, 5°C/min ramp until 250°C, and 250°C for 5 min. Data were recorded in a 5975B mass spectrometer in a 35–250 m/z range at 6.2 scans/s, with an electronic impact ionization set at 70 eV. Chromatograms were analysed using the Enhanced ChemStation E.02.02 software (Agilent Technologies). Identification of the compounds was done by comparing retention times and mass spectrum data with those of standards. In absence of standards, tentative identification was done based on mass spectrum similarities in the NIST05 Mass Spectral Library. For quantitation, a specific ion, having the highest signal-to-noise ratio and being specific enough to provide good peak integration in the specific region of the chromatogram was selected for each compound. A 500 mg aliquot of an admixture reference sample, which was prepared by mixing thoroughly equal amounts of each sample, was analyzed for every six to seven samples and processed as part of the injection series. This admixture was used as a reference to normalize for temporal variation and fiber aging. Finally, the normalized results for a sample were expressed as the ratio of the abundance of each compound in a specific sample to those present in the admixture.

### Untargeted metabolomic analyses by LC-HRMS

Two biological replicates and two technical replicates were processed and analyzed independently. For polar metabolites 10 mg of freeze-dried fruit powder was extracted with 0.75 mL cold 75% (v/v) methanol with 0.5 mg/L formononetin as the internal standard, as previously described [56], and for non-polar analyses 3 mg of powder was extracted with 0.25 mL cold 100% (v/v) methanol, 1 mL of CHCl_3_ with 10 mg/L α-tocopherol acetate as the internal standard, and 0.25 mL of the 50 mM Tris buffer (pH 7.5, with 1 M NaCl) as described previously [57]. LC-HRMS conditions were as previously reported [56]. Polar and apolar metabolomic fractions were prepared as reported above for targeted analysis of the apocarotenoids and carotenoids, respectively. Untargeted analyses were carried out as described previously [56] by using the software SIEVE (Thermofisher scientific). Briefly, after performing sequential in batch alignments, differentially accumulated metabolites (DAMs) in each line compared to the wild-type were identified and used for PCAs. For metabolite identification, different public metabolomic databases (HMD, KEGG, PlantCyc, and Golm Metabolome Database) were searched to retrieve tentative IDs, which were further validated through MS fragmentation, literature search and standard confirmation, when available.

### mRNA expression studies

RNA was extracted from 100 mg of fruits, with the Direct-Zol RNA MicroPrep kit (Zymo Research). Synthesis of the cDNA from each transgenic line and WT was performed using oligo-dT and the Ready-To-Go You-Prime First-Strand Beads (Ge Healthcare). qPCR was carried out using the primers listed in Table S2 and the GoTaq® qPCR Master Mix (Promega, Madison, WI, USA). The constitutively expressed Actin-2 gene transcript was used as a reference gene [58]. The method was as follows: initial denaturation at 94°C for 5 min; 30 cycles of denaturation at 94°C for 20 s, annealing at 60°C for 20 s, and extension at 72°C for 20 s; and a final extension at 72°C for 5 min. The reactions were done in a StepOne™ Thermal Cycler (Applied Biosystems,) and the results analyzed using the StepOne software v2.0 (Applied Biosystems). Three biological and two technical replicas were used for the expression analyses.

### 2,2-Diphenyl-1-picrylhydrazyl (DPPH) radical scavenging activity

Free radical scavenging (FRS) activity was determined as previously described [30]. Fruit samples (50 mg lyophilized fruits) were each extracted with acetone and mixed with 0.2 mM methanolic DPPH. Equal volumes of tomato fruit extract and DPPH solution were mixed and kept in the dark for 30 min at room temperature. The absorbance of the reactions was measured at 517 nm. The FRS was calculated by % = (A_0_-A_1_/A_0_) × 100.

### *C. elegans* assays

Wild-type and transgenic tomato were independently crushed in a blender, the obtained juice was centrifuged at 9000 g for 15 minutes. The liquid phase was collected and lyophilized. The lyophilized of wild-type and transgenic extracts were weighed, resuspended in distilled water, and diluted at six different concentrations (0.04, 0.4, 0.4, 4, 10, and 20 mg of extract/ml), containing 0.1, 0.5, 1, 10, 25 and 50 μg crocins/ml, respectively. *Ginkgo biloba* leaf extract (EGb761) was used as positive control at a concentration of 100 μg/ml. EGb761 contains 24% of flavonoid fraction (composed mainly of quercetin, kaempferol, and isorhamnetin) and 6% of terpenoid fraction (primarily contains ginkgolides A, B, C, J, and M, and bilobalide) [25]. The transgenic *C. elegans* CL4176 strain was used to carry out the experiment. This strain expresses the human amyloid β-peptide 1–42 (Aβ1–42) peptide at 25°C. The worms were synchronized at 16°C in NGM media (negative control) or NGM media supplemented with the positive control or the different dilutions of non-transgenic or transgenic tomato extracts. The temperature was increased to 25°C to activate the Aβ1–42 expression, and the percentage of paralyzed worms was scored at different times (20, 24, 26, 28, 30, and 32 hours). A control without induction was included (NGM without induction). A total of two replicas were performed for each extract and dosage.

### Statistics

For the study of the plant material, three to five biological replicates with three technical replicates per biological replicate were analyzed for every experiment. The obtained data were statistically analyzed with one-way analysis of variance and Duncan’s test of significance using the SPSS software. Levels of significance were determined by t-test. For apocarotenoid data, an ANOVA plus pairwise Tukey’s t-test analysis was performed, by using PAST 4 (https://www.nhm.uio.no/english/research/infrastructure/past/).

## Acknowledgements

This work was supported by grants BIO2016-77000-R from the Spanish Ministerio de Ciencia, Innovación y Universidades and SBPLY/17/180501/000234 from the Junta de Comunidades de Castilla-La Mancha (co-financed European Union FEDER funds) and HARNESSTOM, contract number 101000716 Innovation Action EC-H2020-SFS-2020-1. GD and LGG are participants of the European COST action CA15136 (EUROCAROTEN). GD and AG are participants of the European COST action CA18210 (ROXY). LGG is a participant of the CARNET network (BIO2015-71703-REDT and BIO2017-90877-RED). JLR was supported by the Spanish Ministry of Economy and Competitiveness through a “Juan de la Cierva-Formación” grant (FJCI-2016-28601). The UCLM and the IBMCP researchers constitute the Associated Unit of CAROTENOID BIOTECHNOLOGY.

## Author contributions

Conceptualization: O.A. and L.G.G., writing, review and editing: L.G.G., O.A., A.G., J.L.R. and G.D., plasmids construction: O.A. and A.R-M., plant transformation: S.P., expression analyses: L.G.G. and J.A., metabolites extraction and analyses: L.G.G., G.D., S.F., and J.L.R, antioxidant activities: A.R-M and J.A. plants genotyping: O.A. and J.A. Activity assays: M.L and A.G.

## Data availability

The data supporting our findings are available in the manuscript file or from the corresponding author upon request.

## Conflict interests statement

The authors declare no competing interest.

## Supplementary data

Supplementary data is available at *Horticulture Research* online.

## Supplementary Material

Web_Material_uhac074
