# Supplementary material for: Engineering high levels of saffron apocarotenoids in tomato
Source: Hortic Res. 2022 Mar 23;9:uhac074. doi: 10.1093/hr/uhac074 (PMC9157650; doi:10.1093/hr/uhac074)
Supplement: Web_Material_uhac074 [file web_material_uhac074.zip › Table_S1.docx]

Supplemental Table S1. Oligonucleotides used for plasmids construction.

| **Primers** | **Sequence 5´-3´** |
| --- | --- |
| pUPD2-Dom-UGT2-F | GCGCCGTCTCGCTCGAATGTTGAACGGCAACAAATGC |
| pUPD2-Dom-UGT2-R | GCGCCGTCTCGCTCAAAGCTTAAACTAAGGAAATTTTGGAGTCAT |
| pUPD2-Dom-CsCCD2-L-F1 | GCGCCGTCTCGCTCGAATGGAATCTCCTGCTACTAAATTA |
| pUPD2-Dom-CsCCD2-L-R1 | GCGCCGTCTCGTTGTCTCTGCCTCCTCCTTA |
| pUPD2-Dom-CsCCD2-L-F2 | GCGCCGTCTCGACAAGTAAGAAGAAGCCCAAAC |
| pUPD2-Dom-CsCCD2-L-R2 | GCGCCGTCTCGCTCAAAGCTCATGTCTCTGCTTGGTGCT |
| pUPD2-Dom-UGT709-F | GCGCCGTCTCGCTCGAATGGCTGAGAAAGAAGCAAATAC |
| pUPD2-Dom-UGT709-R | GCGCCGTCTCGCTCAAAGCTCAGGTCCGAAGAAAATTTGG |
