# Supplementary material for: Engineering high levels of saffron apocarotenoids in tomato
Source: Hortic Res. 2022 Mar 23;9:uhac074. doi: 10.1093/hr/uhac074 (PMC9157650; doi:10.1093/hr/uhac074)
Supplement: Web_Material_uhac074 [file web_material_uhac074.zip › Table_S2.docx]

Supplementary Table S2. Oligonucleotides used for expression analyses.

| Gene name | Oligonucleotide forward 5ʹ-3ʹ | Oligonucleotide reverse 5ʹ-3ʹ | Gene |
| --- | --- | --- | --- |
| CsUGT2 | tcgagcctagtgatctgccgt | tcgagccagtccaagtaggga | AY262037 |
| UGT709G1 | actcacctccacgtccttccag | cttgatcagccacgacacaag | KX385186.1 |
| CsCCD2L | ACATGTCGCCTTGAGAGTCC | TCAGATTTGATGCCAGGTTG | KP887110 |
| PSY1 | GGCAATATATGTATGGTGCAGAG | CGCCCATTGAAAACATCTTCA | Solyc03g031860 |
| PSY2 | GTTGATATTCAGCCATTCAGAGAT | TTCAGGTGCAATGCCCATAA | Solyc02g081330 |
| LCYB | TTGGTGGGAATTCAGGGATAGT | AGGCCACAAACCATTCCAAACT | Solyc06g074240 |
| BCH1 | TTGGTGCTGCTGTAGGAATG | GCAATGAGGCCTTTATGGAA | Solyc06g074240 |
| Actin-2 | cattgtgctcagtggtggttc | tctgctggaaggtgctaagtg | Solyc11g005330.2.1 |
