# Supplementary figures and images for: Engineering high levels of saffron apocarotenoids in tomato
Source: Hortic Res. 2022 Mar 23;9:uhac074. doi: 10.1093/hr/uhac074 (PMC9157650; doi:10.1093/hr/uhac074)

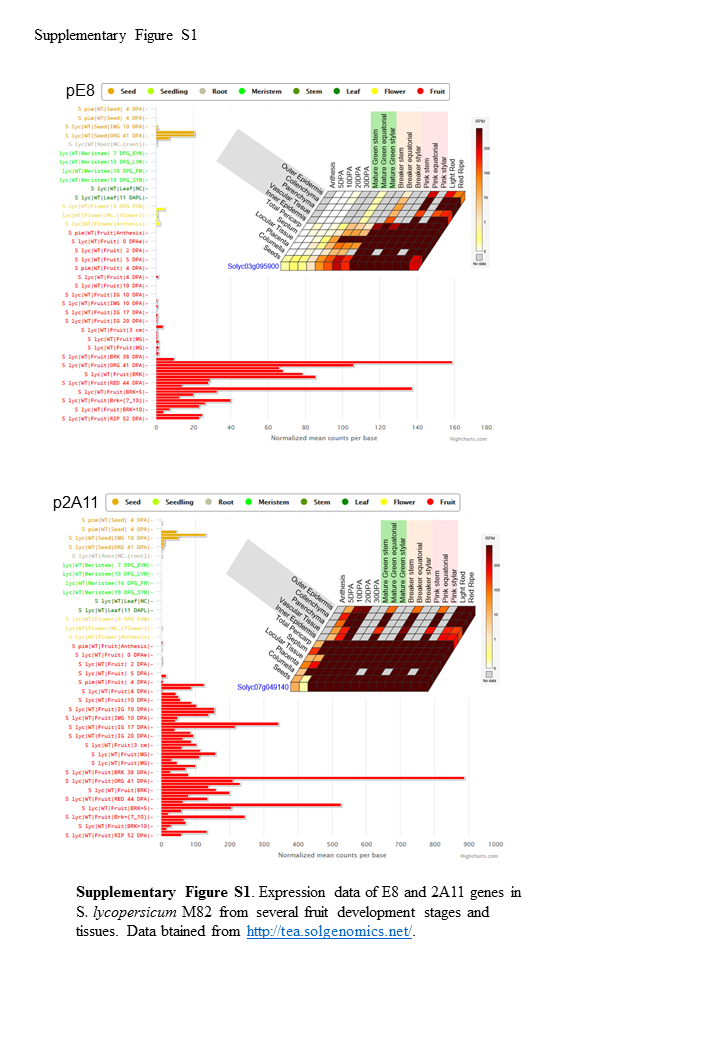

Supplement: Web_Material_uhac074 [file web_material_uhac074.zip › s1.TIF]

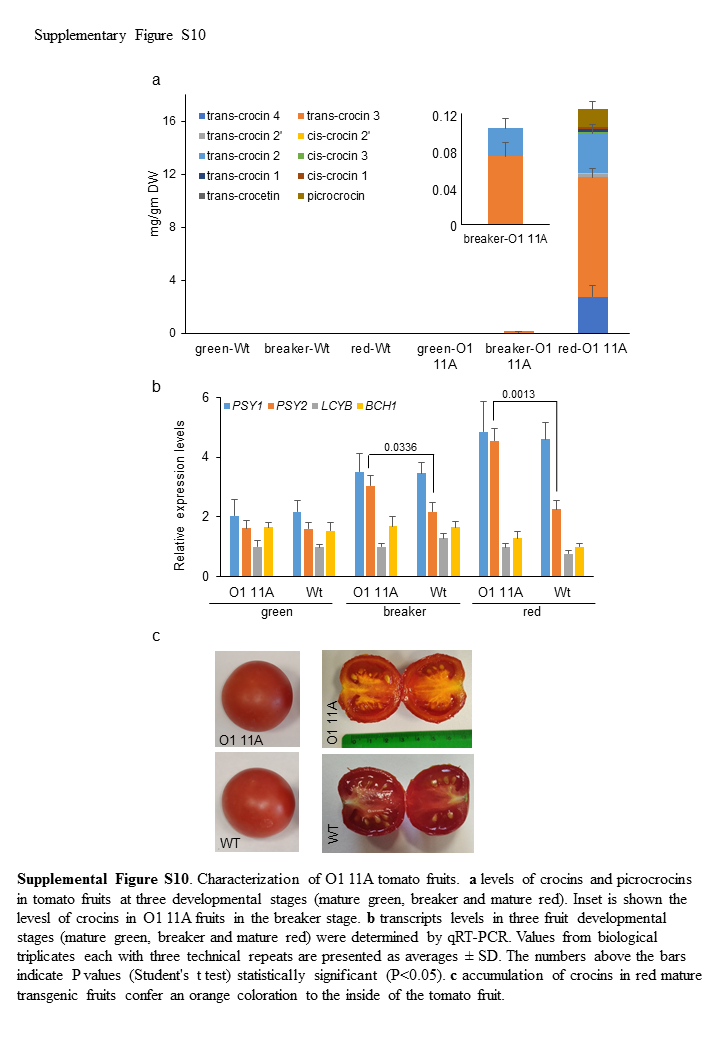

Supplement: Web_Material_uhac074 [file web_material_uhac074.zip › s10.TIF]

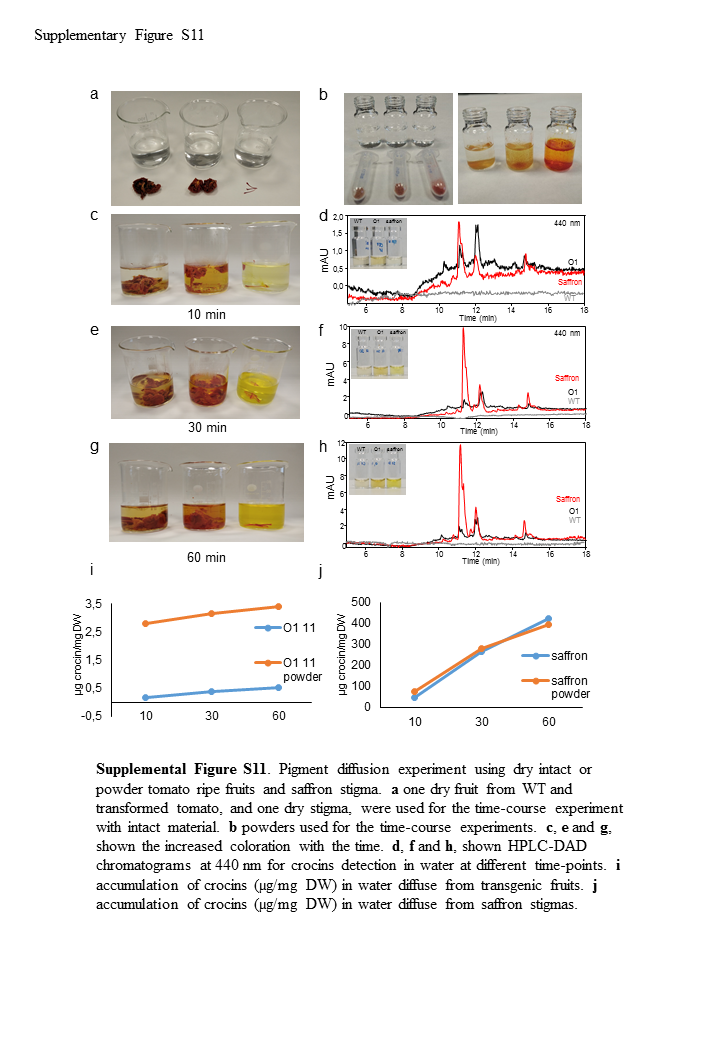

Supplement: Web_Material_uhac074 [file web_material_uhac074.zip › s11.TIF]

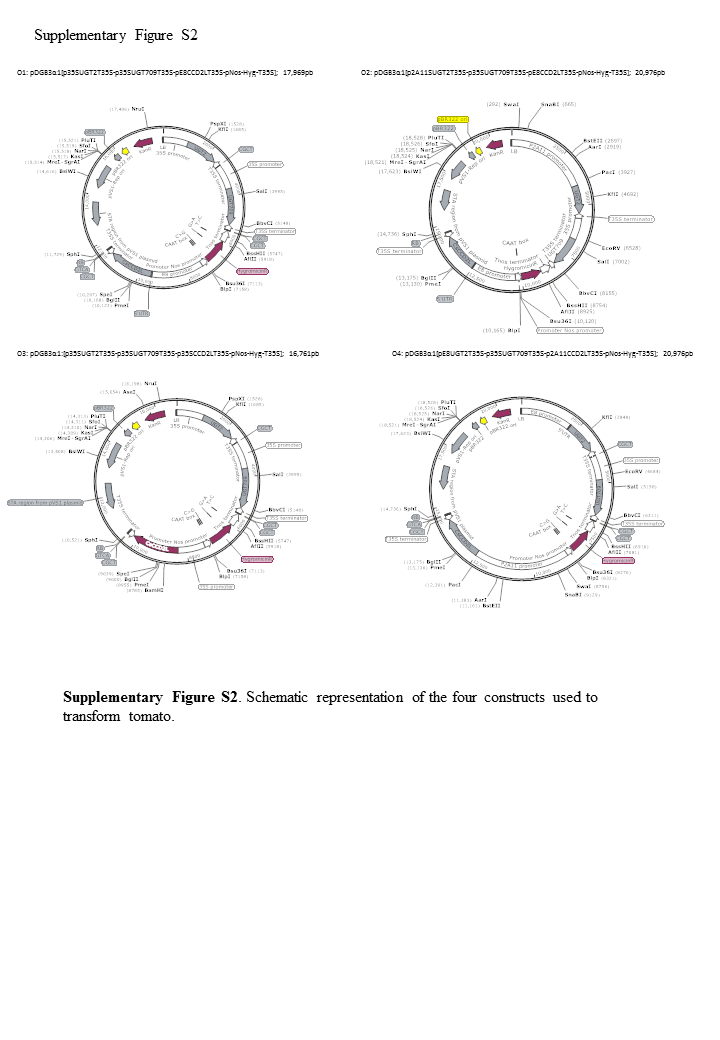

Supplement: Web_Material_uhac074 [file web_material_uhac074.zip › s2.TIF]

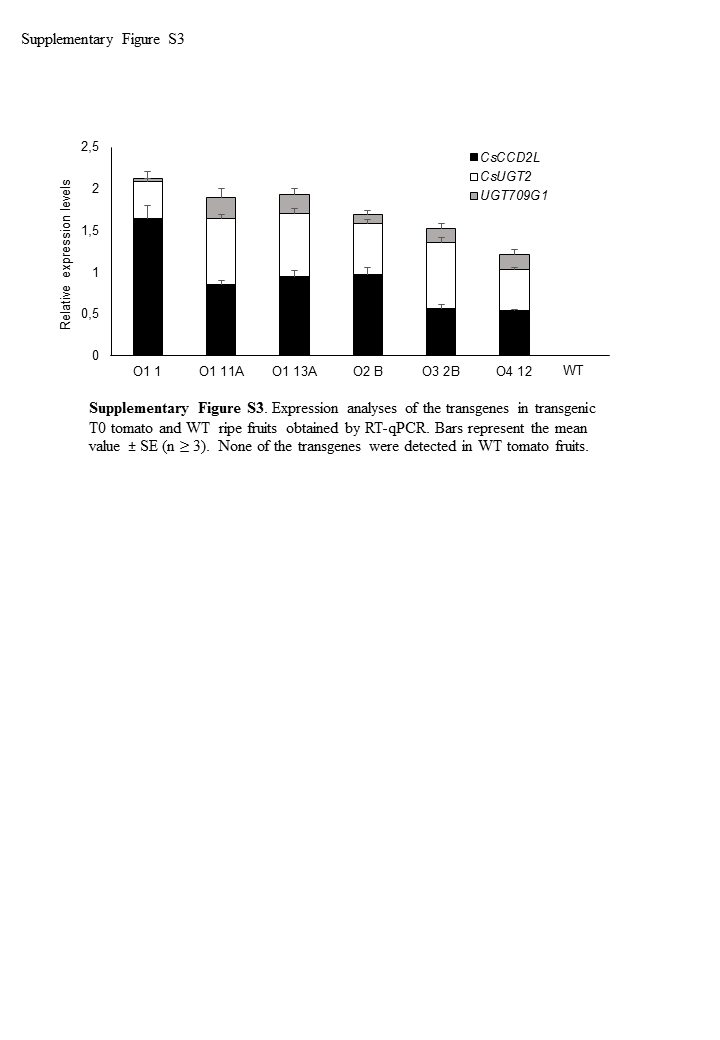

Supplement: Web_Material_uhac074 [file web_material_uhac074.zip › s3.TIF]

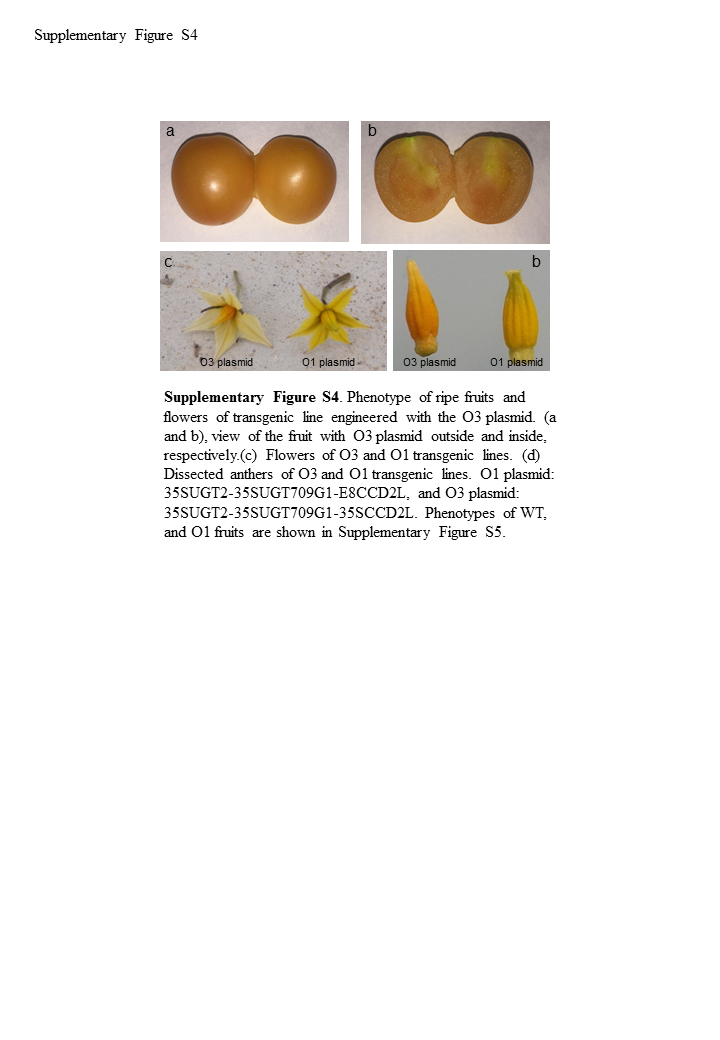

Supplement: Web_Material_uhac074 [file web_material_uhac074.zip › s4.TIF]

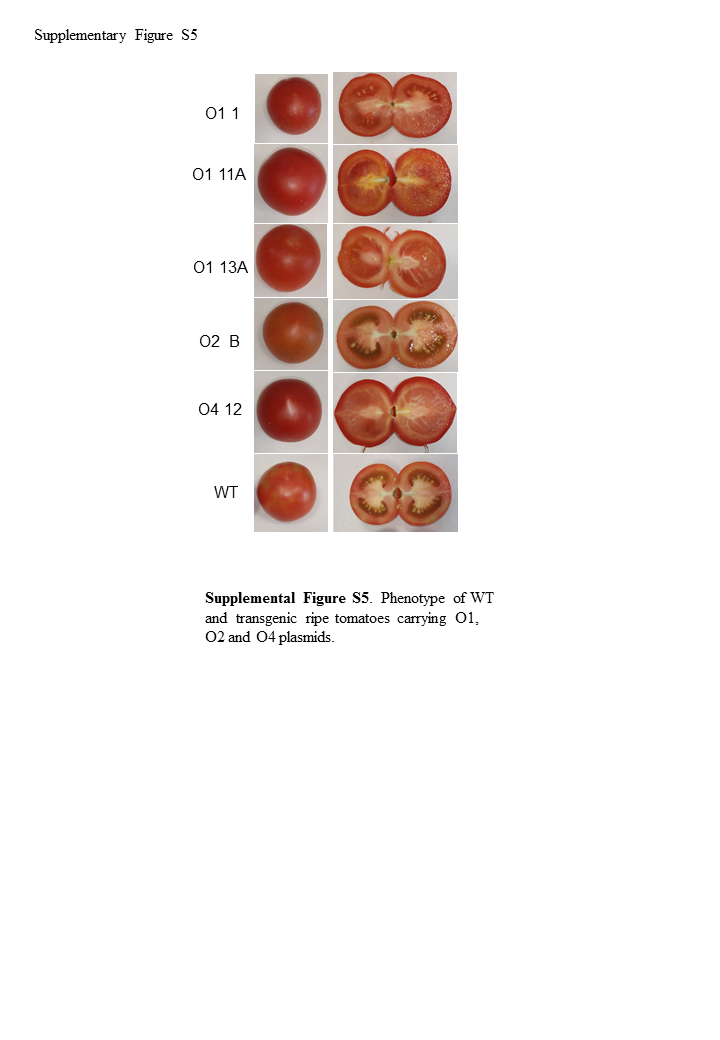

Supplement: Web_Material_uhac074 [file web_material_uhac074.zip › s5.TIF]

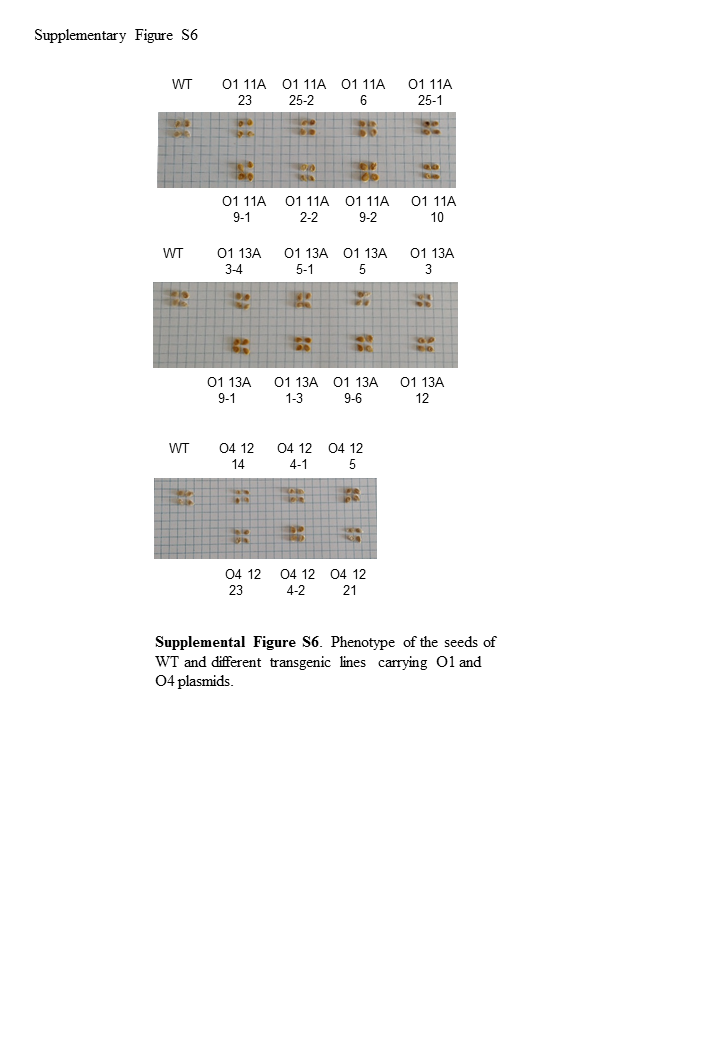

Supplement: Web_Material_uhac074 [file web_material_uhac074.zip › s6.TIF]

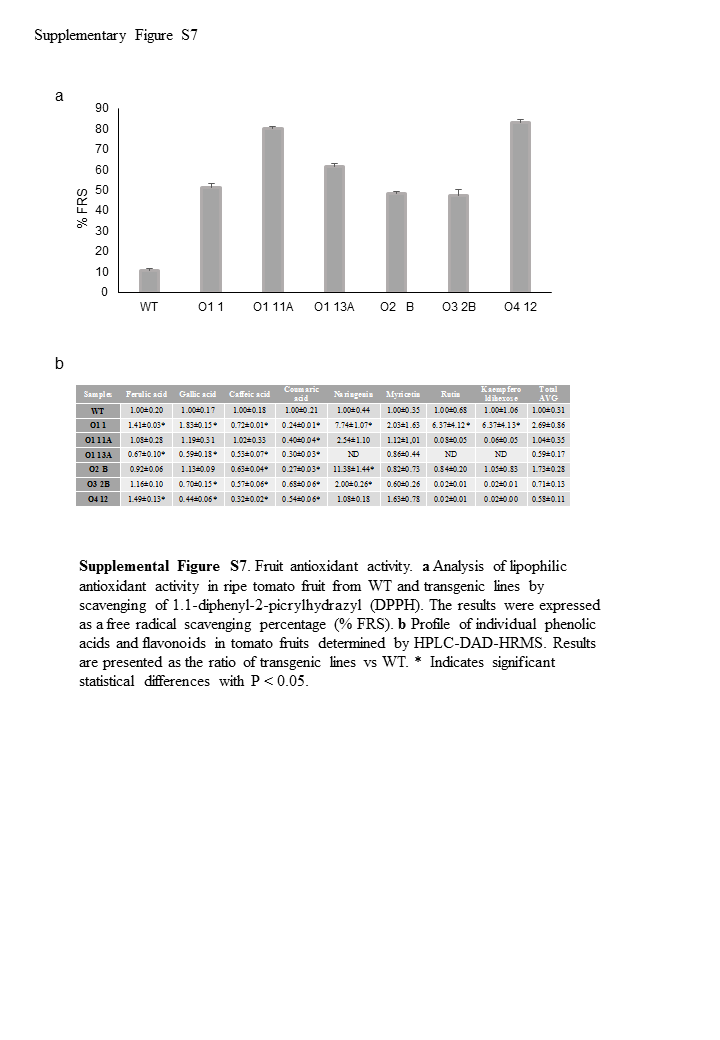

Supplement: Web_Material_uhac074 [file web_material_uhac074.zip › s7.TIF]

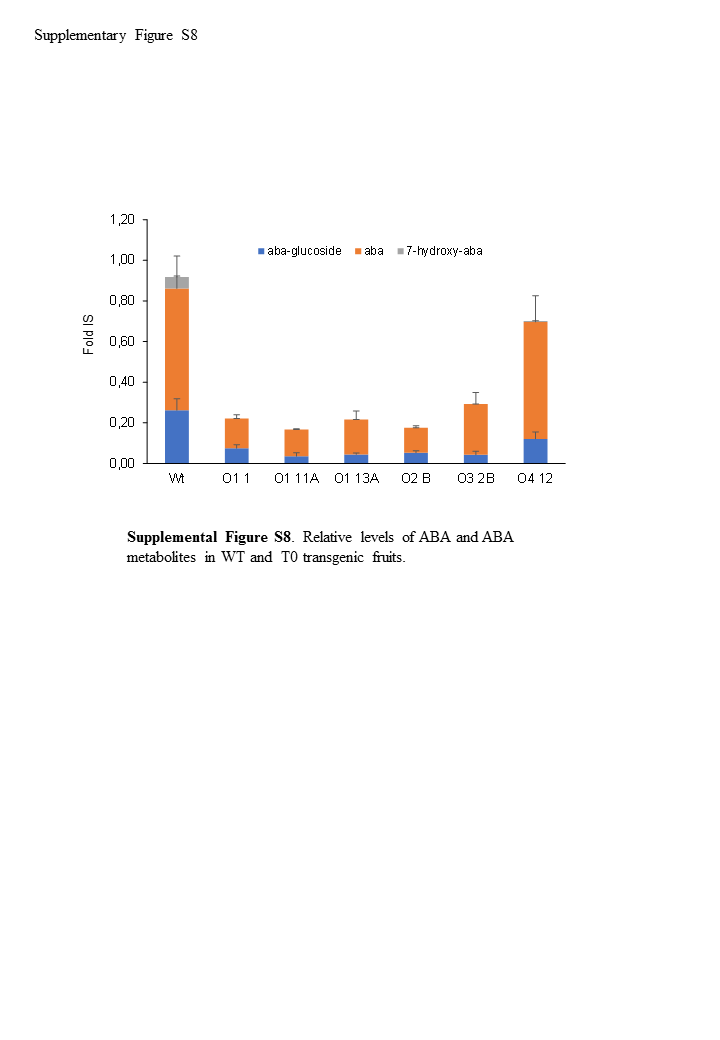

Supplement: Web_Material_uhac074 [file web_material_uhac074.zip › s8.TIF]

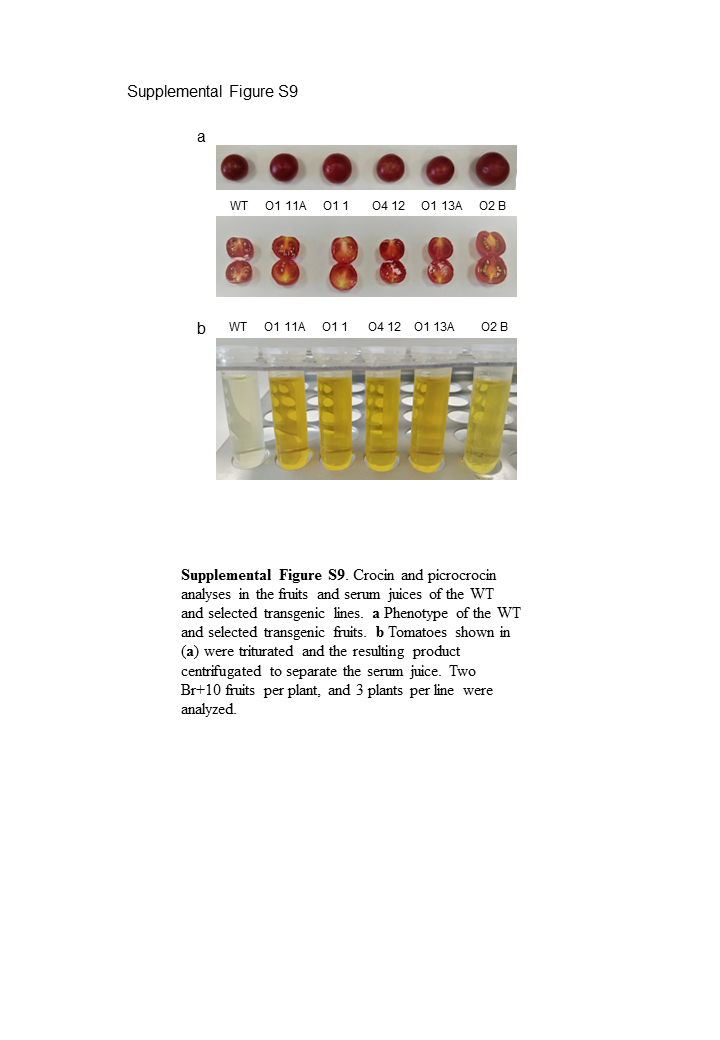

Supplement: Web_Material_uhac074 [file web_material_uhac074.zip › s9.TIF]
